# Immunohistochemistry‐based molecular subtypes of urothelial carcinoma derive different survival benefit from platinum chemotherapy

**DOI:** 10.1002/2056-4538.70017

**Published:** 2025-01-16

**Authors:** Csilla Olah, Oleksandr Shmorhun, Gilbert Georg Klamminger, Josefine Rawitzer, Lara Sichward, Boris Hadaschik, Mulham Al‐Nader, Ulrich Krafft, Christian Niedworok, Melinda Váradi, Peter Nyirady, Andras Kiss, Eszter Szekely, Henning Reis, Tibor Szarvas

**Affiliations:** ^1^ Department of Urology University of Duisburg‐Essen Essen Germany; ^2^ Dr. Senckenberg Institute of Pathology University Hospital Frankfurt, Goethe University Frankfurt Frankfurt am Main Germany; ^3^ Institute of Pathology University Medicine Essen, University of Duisburg‐Essen Essen Germany; ^4^ Department of Urology Hermann‐Josef‐Hospital Erkelenz Germany; ^5^ Department of Urology Semmelweis University Budapest Hungary; ^6^ Department of Pathology, Forensic and Insurance Medicine Semmelweis University Budapest Hungary

**Keywords:** bladder cancer, molecular subtype classification, cisplatin, chemotherapy, immunohistochemistry, Lund Taxonomy

## Abstract

Distinct molecular subtypes of muscle‐invasive bladder cancer (MIBC) may show different platinum sensitivities. Currently available data were mostly generated at transcriptome level and have limited comparability to each other. We aimed to determine the platinum sensitivity of molecular subtypes by using the protein expression‐based Lund Taxonomy. In addition, we assessed the tumor heterogeneity within the primary tumor and between the primary and lymph node (LN) metastatic sites. Thirteen immunohistochemical markers were stained in a tissue microarray with an overall number of 1,508 cores. Statistical evaluation was performed in 199 patients divided into three chemo‐naïve MIBC cohorts: (1) pT3/4 and/or LN+ patients who received radical cystectomy without platinum treatment, (2) patients who received adjuvant chemotherapy (AC), and (3) patients who underwent palliative platinum treatment for metastatic disease or postoperative progression. Overall survival (OS) was used as the primary endpoint. Patients with the genomically unstable (GU) subtype had significantly better OS in the AC group compared to the radical cystectomy group (HR: 0.395, 95% CI: 0.205–0.795, *p* = 0.005). In contrast, no such association was observed for the basal/squamous (Ba/Sq) subtype. Intratumor heterogeneity was present in 19% of cases, with the lowest level in the Ba/Sq and GU tumors (14% each) and the highest level of 43% in small‐cell/neuroendocrine‐like tumors. There was greater subtype heterogeneity between primary tumors and LN metastases. In conclusion, immunohistochemistry‐based Lund Taxonomy subtypes remain stable within the same primary tumor, with the GU subtype deriving the greatest OS benefit from AC. However, high tumor heterogeneity between the primary tumor and metastatic sites can impact the effectiveness of therapies.

## Introduction

Previous studies have revealed repeatedly occurring distinct gene expression profiles in muscle‐invasive bladder cancer (MIBC), leading to the definition of molecular subtypes [1, 2]. The existence of these subtypes may potentially explain the large heterogeneity observed in MIBC patients' response to surgical, systemic, or targeted therapies. Radical cystectomy (RC) with pelvic lymph node (LN) dissection is the gold standard surgical treatment for MIBC, but it yields only 40–60% 5‐year survival rates. Therefore, perioperative platinum‐based chemotherapy is recommended. Neoadjuvant chemotherapy (NAC) is advised for N0M0 MIBC patients, providing an 8% 5‐year survival benefit. Adjuvant chemotherapy (AC) is recommended for patients with pT3/4 or LN‐positive tumors who did not receive NAC, offering a significant 5‐year survival benefit of 6% compared to RC alone [3, 4].

Various molecular subtypes showed different sensitivities to systemic therapies, suggesting their potential use in therapy prediction [5, 6, 7, 8, 9]. However, most studies rely on transcriptome sequencing, a technique that is expensive and requires complex bioinformatic analysis, limiting its feasibility for routine clinical use. Therefore, several immunohistochemistry (IHC)‐based molecular subtype classification methods have been reported with typically two to five markers, which were suggested to identify basal, luminal along with double negative and double positive/mixed groups [10, 11, 12]. However, these methods are unable to identify less frequently occurring molecular subtypes, such as small‐cell/neuroendocrine‐like (Sc/Ne) or those within the luminal subtype, which may hold clinically significant predictive value for chemotherapy (CTx) response. In contrast to these rather limited classifiers, the Lund research group developed an IHC‐based methodology and a step‐by‐step protocol using 13 protein markers, which identifies the same five molecular Lund Taxonomy subtypes as those derived from transcriptome‐based gene expression profiling [13, 14].

In the last few years, new systemic treatments have become available for MIBC patients, including immune checkpoint inhibitors (ICIs) and novel drugs targeting the fibroblast growth factor receptor (FGFR), nectin‐4, or TROP2 molecules. These therapies were initially recommended as second‐ or third‐line options for patients following platinum treatment. Enfortumab vedotin, which targets nectin‐4, has been or is now being investigated in clinical studies for its efficacy in the first‐line metastatic and neoadjuvant settings [15]. The molecular subtypes also showed different sensitivities to ICIs [16, 17], and thus may have an important role in therapeutic decision‐making in the future. The ongoing prospective, multicenter GUSTO (Gene Expression Subtypes of Urothelial Carcinoma: Stratified Treatment and Oncological Outcomes) trial, is currently investigating the utilization of molecular subtypes in the perioperative MIBC setting [18]. Overall, considerable attention is focused on the clinical implementation of molecular subtypes; however, the development of a simpler and cost‐effective subtyping method is crucial for its integration into the daily routine.

In the present study, we aimed to apply the IHC‐based Lund Taxonomy, identifying five subtypes, to assess its prognostic and platinum‐predictive value in a large consecutive RC cohort with or without CTx.

## Materials and methods

### Tissue microarray cohort and tissue collection

**Figure 1:**
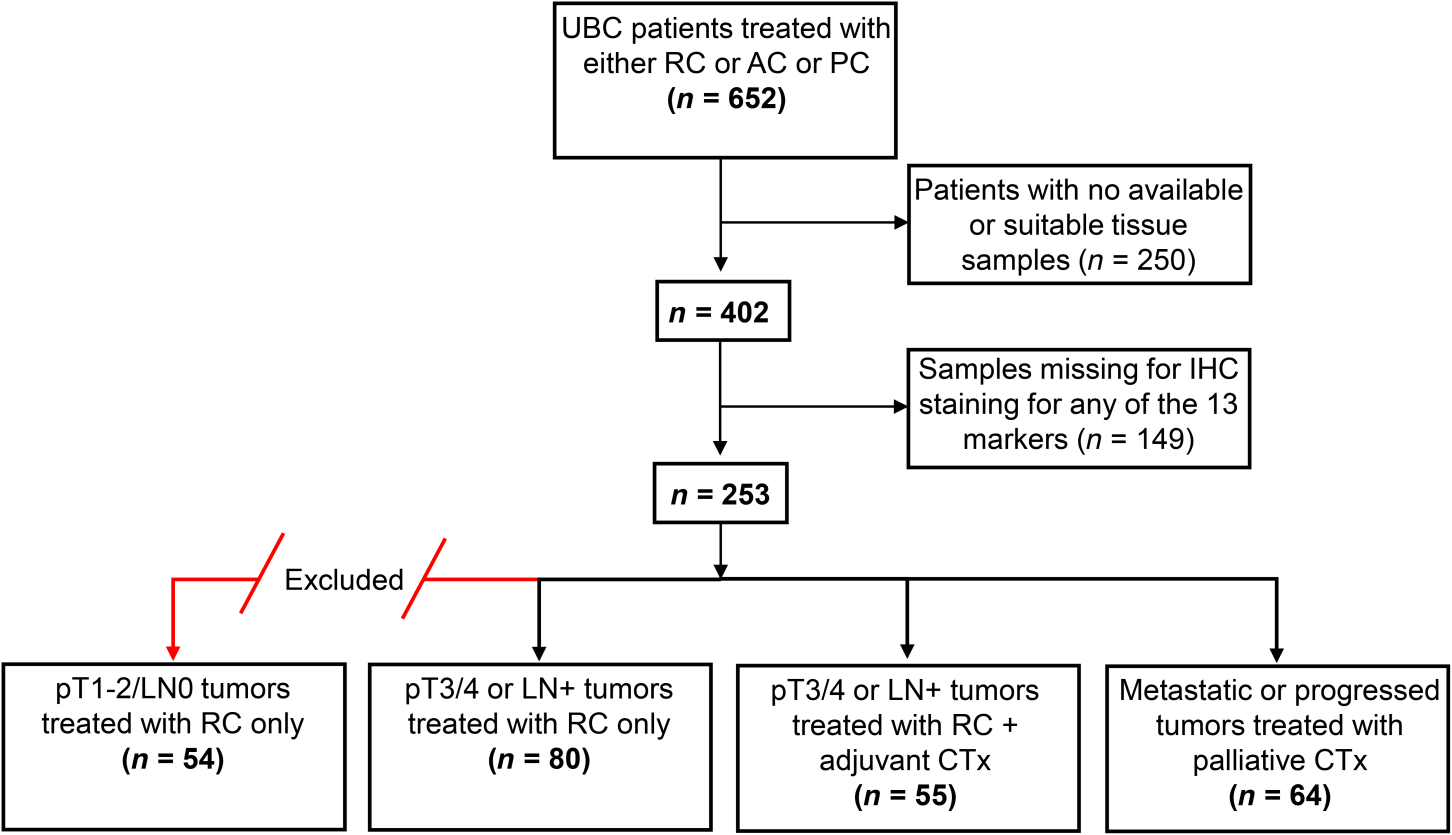
Overview of patient cohorts with details on exclusion. AC, adjuvant chemotherapy; CTx: chemotherapy; IHC, immunohistochemistry; PC, palliative chemotherapy; RC, radical cystectomy; UBC, urothelial bladder cancer.

This study included a total of 652 urothelial MIBC patients, who underwent RC at the Department of Urology, University of Duisburg‐Essen, between 2005 and 2018, or participated in a phase‐II, prospective, multicenter, randomized, double‐blinded trial (SUSE, AB 31/05, RUTT 204) and received postoperative CTx [19]. Patients with unavailable or insufficient formalin‐fixed and paraffin‐embedded (FFPE) samples were excluded from the study (*n* = 250). IHC‐based molecular subtypes were successfully determined for 253 patients (only those cases with all 13 IHC stainings available). As we focused on patients with indication for CTx, we excluded 54 patients with pT1‐2/LN0 tumors, who underwent RC, but had no indication for postoperative CTx. Subsequently, three subcohorts were defined for the final analysis; (1) patients with pT3/4 or LN+ tumors (present with AC indication), who underwent solely RC without CTx (*RC cohort*); (2) patients with pT3/4 or LN+ tumors, treated with RC and CTx within 90 days after surgery (*AC cohort*); and (3) patients treated with palliative CTx (*PC cohort*). Patients in the PC cohort received CTx after more than 90 days following RC because of postoperative progression or were ineligible for surgery (Figure 1). The research received approval from the institutional ethics committee (15‐6400‐BO) and adhered to the principles outlined in the Declaration of Helsinki.

From the FFPE samples, a tissue microarray (TMA) was constructed with 2‐mm diameter tissue cores. Simultaneously several cores were punched from each case as follows: two from the tumor central (TC) region, two from the tumor‐normal interface (TNI) region, and two from positive LNs (if present). This sampling method enables the examination of intra‐ and intertumoral heterogeneity of various proteins and the molecular subtypes. Patients' clinicopathological as well as follow‐up data, such as radiographic progression‐free survival (rPFS) and overall survival (OS) were available. The prognostic value of molecular subtypes was analyzed in each subcohort (RC, AC, and PC). The CTx predictive value of subtypes was directly investigated comparing patients' outcomes between the RC and AC cohorts in each molecular subtype group. For this evaluation, we considered only those patients with indication for AC (pT3/4 and/or LN+) and stratified them for OS to those who received (AC cohort) versus did not receive AC (RC cohort) in each molecular subtype group.

### IHC analysis and molecular subtyping

Thirteen protein markers were stained and evaluated based on the Molecular Taxonomy method as described by the Lund research group [14]. Supplementary material, Table S1 summarizes the applied antibodies along with accession numbers and evaluation methods. From the TMA blocks, unstained sections of 3–4 μm were freshly cut and stained on automated IHC devices following the manufacturers' recommendations (Ventana Benchmark Ultra, Roche/Ventana, Oro Valley, AZ, USA; Dako Omnis, Dako/Agilent, Santa Clara, CA, USA). The IHC staining patterns characteristic of the various molecular subtypes, along with the protein expression results of the 13 markers and the different subtype scores calculated from the protein expression data, are illustrated in supplementary material, Figures S1 and S2. Further details of the IHC staining, protein expression evaluation and raw staining results of the 13 markers are provided in Supplementary methods and results and supplementary material, Table S1.

### Statistical analysis

Chi‐square test was used to examine the correlation between clinicopathological parameters and molecular subtypes using the basal/squamous (Ba/Sq) subtype as the reference. Mann–Whitney test for continuous clinical variables and chi‐square test for dichotomized variables were used to test the associations between different treatment cohorts. Cox univariable analysis was conducted to assess OS and rPFS, and additional multivariable analysis was performed with the inclusion of those parameters that reached the *p* value of ≤0.05 in the univariable analysis. OS and rPFS were calculated from treatment start to death/progression or censoring at last follow‐up (June 2021). Kaplan–Meier plots were used to visualize survival differences between molecular subtypes as well as between the RC and AC cohorts in different subtype groups. Sankey plots were generated in R to visualize molecular subtype's concordance between different tumor areas (TC versus TNI) (R version 4.2.1, Vienna, Austria). Statistical analyses were conducted using the SPSS software package (IBM SPSS Statistics for Windows, version 25, IBM Corp., Armonk, NY, USA). All statistical tests with a *p* value of ≤0.05 were considered significant.

## Results

### Cohort description

The final analysis included a total of 199 patients stratified into three different subcohorts based on the treatment setting: (1) solely RC‐treated patients (*n* = 80) with AC indication (pT3/4 or LN+ disease), who refused or were ineligible for AC; (2) AC‐treated patients (*n* = 55); and (3) PC‐treated patients (*n* = 64). The main characteristics of these cohorts are provided in Table 1. Patients in the RC cohort had a significantly higher age at surgery (*p* < 0.001), while other clinicopathological characteristics (e.g. stage distribution, male/female ratio) were similar between the RC and AC groups.

**Table 1:** Patient characteristics

Variables		Whole cohort, *n* (%)	Control	Chemotherapy	
			RC cohort, *n* (%)	AC cohort, *n* (%)	PC cohort, *n* (%)
Total number of patients		199	80	55	64
Age at baseline median (range)		68 (39–90)	74 (39–90)	62 (41–82)	65 (39–90)
Sex	Male	145 (73)	62 (78)	37 (67)	46 (72)
	Female	54 (27)	18 (23)	18 (33)	18 (28)
Cystectomy data	pT2	25 (13)	3 (4)	8 (15)	14 (22)
	pT3	102 (51)	55 (69)	31 (56)	16 (25)
	pT4	49 (25)	22 (28)	16 (29)	11 (17)
	Not available	23 (12)	0	0	23 (36)
Metastases	LN0	122 (61)	47 (59)	27 (49)	48 (75)
	LN+	70 (35)	28 (35)	27 (49)	15 (23)
	Not available	7 (4)	5 (6)	1 (2)	1 (2)
	M0	127 (64)	50 (63)	45 (82)	32 (50)
	M+	28 (14)	0	0	28 (44)
	Not available	44 (22)	30 (38)	10 (18)	4 (6)
Chemotherapy regimen	Gem/Cis	101 (85)	–	53 (96)	48 (75)
	Carboplatin	4 (2)	–	2 (4)	2 (3)
	Cis/MTX	6 (3)	–	0	6 (9)
	MVAC	8 (4)	–	0	8 (13)
Number of patients who died		151 (76)	64 (80)	36 (65)	51 (80)
Follow‐up time in months median (range)		16 (1–172)	8 (1–163)	27 (2–157)	18 (1–114)
Number of patients who progressed after chemotherapy		50 (25)	–	18 (33)	32 (50)
Follow‐up time in months median (range)		–	–	12 (1–127)	4 (1–102)
Subtype class information (tumor central)					
Lund Taxonomy	Urothelial‐like	33 (17)	15 (19)	8 (15)	10 (17)
	Genomically unstable	98 (49)	45 (56)	22 (40)	31 (48)
	Basal/squamous	46 (23)	18 (23)	17 (31)	11 (17)
	Mesenchymal‐like	6 (3)	2 (3)	1 (2)	3 (5)
	Small‐cell/neuroendocrine‐like	16 (8)	0	7 (13)	9 (14)

AC, adjuvant chemotherapy; Cis, cisplatin; Gem, gemcitabine; MVAC, methotrexate, vinblastine sulfate, doxorubicin hydrochloride and cisplatin; MTX, methotrexate; PC, palliative chemotherapy; RC, radical cystectomy.

The distribution of molecular subtypes was comparable across the various treatment groups, with the genomically unstable (GU) subtype being the most prevalent in each cohort (Table 1). According to previously published literature, a significantly higher rate of females was observed in the Ba/Sq tumors (41%) compared to the urothelial‐like (Uro) and GU subtypes (20%, *p* = 0.042; 21% *p* = 0.013, respectively). Additionally, patients with Ba/Sq tumors had a higher pathological stage (pT3‐4; 93%) than those with Sc/Ne (pT3‐4; 73%, *p* = 0.043) and Uro (80%, *p* = 0.089) subtypes; however, this latter association did not reach the significance level (supplementary material, Table S2).

### Survival analysis with clinical parameters and molecular subtypes

In the univariable analysis, Uro and GU subtypes were associated with improved OS compared to the Ba/Sq subtype in the PC cohort (*p* = 0.001 and *p* = 0.001, respectively). Consistent with these findings, the Uro and GU subtypes were associated with longer rPFS in the PC cohort (*p* = 0.011 and *p* = 0.034), while the GU subtype was also associated with longer rPFS in the AC cohort (*p* = 0.014) (supplementary material, Table S3A,B). Detailed results of the Cox uni‐ and multivariable analysis are presented in Supplementary methods and results.

In the RC cohort, the molecular subtypes were associated with significantly different OS rates (log rank: *p* < 0.001), with the mesenchymal‐like (Mes) subtype demonstrating the worst OS; however, it was present in only 3% of patients. Similarly, in the PC cohort, the different subtypes showed significant differences in OS rates (log rank: *p* = 0.001), whereas in the AC cohort, no significant differences were observed among the various subtypes. Notably, significantly different rPFS were identified in the AC cohort (log rank: *p* = 0.046), with the GU subtype showing the most favorable outcome. Moreover, the Uro and GU subtypes had significantly better OS compared to other subtypes (log rank: *p* < 0.001) and exhibited an improved rPFS within the PC cohort (supplementary material, Figure S3). Overall, in the platinum untreated RC cohort the Ba/Sq subtype had the longest OS, while in the platinum‐treated cohorts the GU and Uro subtypes showed the most favorable OS and rPFS.

### Platinum‐predictive value of molecular subtypes

**Figure 2:**
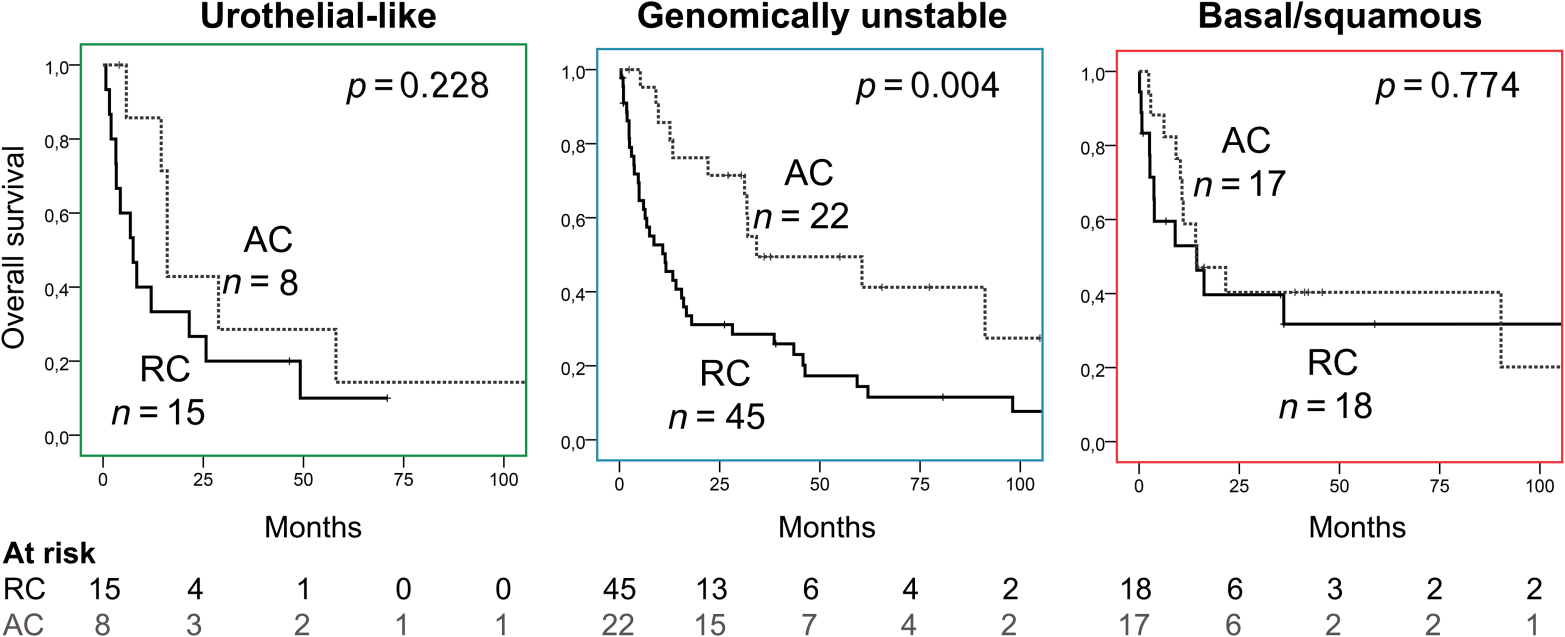
Survival analysis between chemotherapy‐treated (AC) and untreated (RC) patient groups identified with different molecular subtypes. AC, adjuvant chemotherapy; RC, radical cystectomy.

The CTx predictive values of the three largest subtypes (Uro, GU, and Ba/Sq) were directly examined by comparing OS of MIBC patients (all present with AC indication; pT3/4 or LN+). OS was assessed in each molecular subtype group separately between patients who received platinum therapy (AC cohort) and those who did not (RC cohort). This evaluation method enables the identification of those molecular subtypes that potentially benefit from an AC treatment. Patients with the GU subtype exhibited a significantly longer OS in the AC compared to the RC group (HR: 0.395, 95% CI: 0.205–0.795, *p* = 0.005), suggesting that patients with GU tumors derive a significant OS benefit from AC. In contrast, in the Ba/Sq subtype no OS difference could be observed between the AC and RC treatment groups (HR: 0.887, 95% CI: 0.390–2.015, *p* = 0.774), suggesting no OS benefit for the AC treatment in this molecular subgroup (Figure 2). Low case numbers of the additional two less frequently occurring subtypes (Mes and Sc/Ne) precluded a valid statistical evaluation.

While the main clinicopathological characteristics of patients in the RC and AC cohorts were comparable, the age in the RC cohort was higher compared to the AC cohort (median 74 versus 62 years, *p* < 0.001), which may represent a selection bias with an impact on the OS outcome. Therefore, to better address this aspect, we performed OS analysis comparing low and high age groups (cut‐off: 65 years) both in the RC and AC groups (supplementary material, Figure S4A). This analysis revealed no survival differences in either of the treatment groups (RC: log rank *p* = 0.957; AC: log rank *p* = 0.900). Conversely, the application of AC showed a significant survival advantage in both age groups (≤65 years: log rank *p* = 0.008; >65 years: log rank *p* = 0.037) (supplementary material, Figure S4B). These results collectively suggest that the age difference between the two treatment groups (RC and AC) does not meaningfully influence the analysis.

### Testing models with reduced IHC marker sets

While IHC‐based assays with a few markers are easily integrated into the clinical routine, staining for 13 protein markers for molecular subtyping remains challenging. Therefore, Hardy *et al* aimed to simplify the Lund IHC Taxonomy by reducing the marker set using algorithms on the original Lund cohorts and then suggested two simplified models with two and three proteins [20]. We assessed these simplified models for their platinum‐predictive performance in our samples. Overall, the simplified models showed poorer efficacy in differentiating between the GU and Uro subtypes, with 35% (8/23) and 39% (9/23) of Lund Taxonomy Uro samples being classified as GU by model 1 and model 2, respectively, and thereby seemed to have reduced predictive value for platinum‐based CTx (data are presented in Supplementary methods and results and supplementary material, Figure S5).

### Subtype heterogeneity in the primary tumor and between primary versus corresponding LN metastatic sites

**Figure 3:**
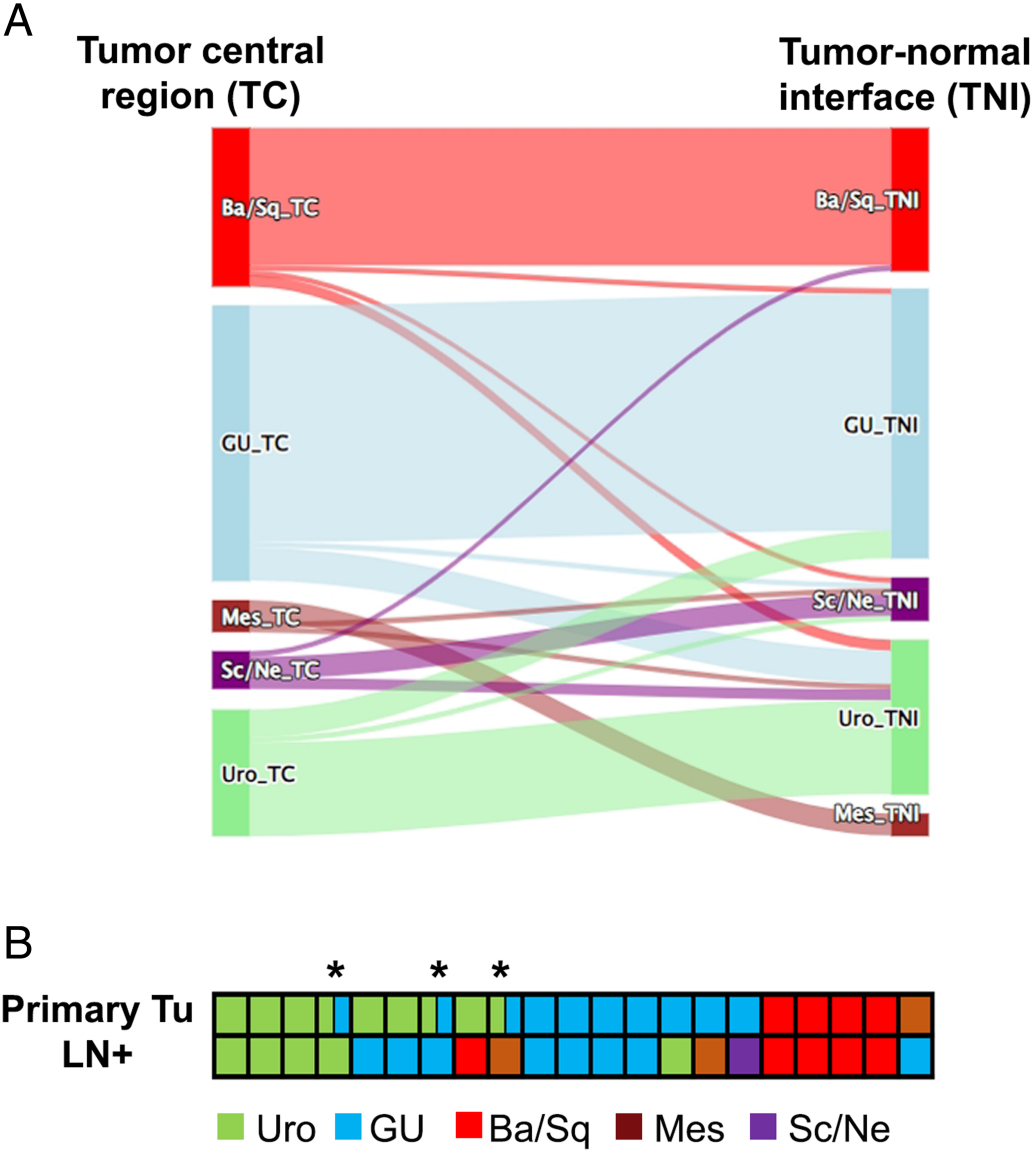
Subtype heterogeneity within (A) tumor central and tumor‐normal interface regions and (B) between primary and LN metastatic sites. Ba/Sq, basal/squamous; GU, genomically unstable; LN+, positive lymph node; Mes, mesenchymal‐like; Sc/Ne, small‐cell/neuroendocrine‐like; TC, tumor central; Tu, tumor; Uro, urothelial‐like. The presence of intratumor heterogeneity within the primary tumors (TC versus TNI) is presented with *.

Intratumor heterogeneity presents a challenge in cancer therapy, as different tumor subclones may not consistently respond to various treatments. Therefore, we examined the presence of molecular subtype differences within two different tumor regions (TC versus TNI) of the same tumor in 115 cases. Molecular subtypes showed a low intratumor heterogeneity, with an overall overlap of 81%. The Ba/Sq and GU subtypes exhibited the highest consistency with an overlap of 86% (each), followed by the Uro subtype of 74%. The most commonly occurring shift was observed between the Uro and GU subtypes. In contrast, the two less frequently occurring subtypes, Mes and Sc/Ne, had the lowest overlaps between TC and TNI, with 67% and 57%, respectively (Figure 3A).

Molecular subtype information was available for 21 primary/LN tumor tissue pairs. Only 12 (57%) of these cases showed consistency. The Ba/Sq subtype exhibited 100% (4/4) overlap between primary and LN metastatic sites, while the GU subtype showed a lower rate of overlap at 57% (4/7), and the Uro subtype had only 44% (4/9) overlap. Additionally, intratumor heterogeneity was observed in three of the nine Uro cases, where the GU subtype was also present (Figure 3B).

## Discussion

In the present study, we performed a 13 IHC marker‐based molecular subtype classification in a large, consecutive MIBC cohort in order to assess (1) the benefit of platinum CTx in different molecular subtypes, (2) the intratumor subtype heterogeneity, and (3) a potential subtype shift between primary tumor and matched LN metastasis. We found that patients with the GU subtype benefited from platinum‐based CTx with improved OS, while those with other subtypes did not show improved OS when receiving platinum treatment. Intratumor heterogeneity was present in only 19% of cases with the lowest level in the Ba/Sq and GU tumors (14% each) and the highest level in Sc/Ne of 43%. In contrast, corresponding LN metastases showed only a 57% subtype overlap with their primary site with higher stability of the Ba/Sq subtype (100%).

In the last few years, several studies examined the CTx‐predictive value of different molecular MIBC subtypes. In the earliest study from the MD Anderson (MDA) Cancer Center, the authors assessed the pathological response to NAC and identified the p53‐like subtype to be platinum resistant [5]. In a later study by Seiler *et al*, using the genomic subtyping classifier (GSC), the basal subtype was shown to derive OS benefit from NAC; however, this could not be confirmed by the pathological response as an endpoint [6]. A subsequent study using the GSC classification method found that the merged nonluminal subtypes had an OS benefit from NAC. However, the pathological response rates of the subtypes were not reported in this study either [21]. A further study identified a survival benefit for patients with Ba/Sq and stroma‐rich Consensus subtypes who received AC compared to those treated with RC alone [9]. However, subsequently, three independent prospective studies could not confirm the predictive value of the basal subtype in the NAC setting [22, 23, 24]. Two Scandinavian studies found that the luminal than the basal subtypes are more responsive to platinum treatment [7, 8]. In accordance, our previous gene expression‐based analysis assessing MIBC patients who underwent AC versus RC‐only therapy found that patients with the luminal but not Ba/Sq subtype derive OS benefit from AC [25]. However, the comparability of all these studies is limited by the different therapy settings (neoadjuvant, adjuvant, inductive CTx), endpoints (pathological response, radiographic response, OS, and cancer‐specific survival) as well as different subtype classification methods (MDA, GSC, Consensus, Lund). The development of a Consensus classification aimed to address the challenges arising from the parallel application of different classification systems in various studies [2]. All of the above studies applied gene expression‐based methodologies, mainly transcriptome sequencing, which is expensive, requires higher amounts of purified RNA and complex data evaluation methodology and thus are less compatible with clinical routine application. Therefore, IHC‐based methods may represent an alternative approach for molecular classification; however, there is much less data available for these methods, mostly with smaller MIBC cohorts.

In the present study, we applied the 13 IHC marker‐based Lund Taxonomy to assess potentially differential platinum sensitivity of subtypes [14]. Our data revealed higher platinum sensitivity of GU compared to the Ba/Sq tumors in the adjuvant setting. In addition, the Uro subtype also showed improved radiographic PFS in our AC and PC cohorts. These data are in accordance with the former observations made in the neoadjuvant setting by using the Lund Taxonomy, showing that the GU and Uro subtypes have greater platinum sensitivity compared to the Ba/Sq subtype [7]. In a subsequent study by the Lund research group involving patients with metastatic urothelial cancer treated with palliative CTx, the authors confirmed a favorable PFS and OS for patients with the Uro (7.7 months PFS, 13.1 months OS) and GU (11.7 months PFS, 15.5 months OS) Lund Taxonomy IHC subtypes. In contrast, patients with the Ba/Sq IHC subtype had shorter outcomes, with 4.4 months PFS and 7.1 months OS [26]. A further study made similar observations in the inductive CTx setting [8]. These data collectively suggest that patients with GU or Uro subtypes derive more benefit from platinum treatment independent of therapy setting. The identified Lund Taxonomy subtypes differ not only in gene and protein expression profiles but also in the composition of their tumor microenvironment. Ba/Sq tumors are characterized by high immune cell infiltration, while the Uro and GU subtypes exhibit a significantly higher proportion of CD4+ T cells and a higher CD4+/CD8+ ratio. A more detailed analysis of CD4+ T cells revealed that the proportion of regulatory T cells was higher in GU tumors compared to Uro tumors [27]. These differences in immune cell composition may influence responses to cancer therapies.

As staining for 13 IHC markers requires much effort, which is hardly compatible with the daily routine, reduction of the number of markers for a simplified subtype classification has been tested [20]. As most of these markers were included in our 13 IHC panel, we could test the predictive performance of the reduced sets. Our data show that these simplified methods provide similar but clinically less relevant information.

Considering the different therapy sensitivities of various MIBC subtypes, the parallel existence of distinct subtypes may represent a significant problem. However, the intratumoral subtype heterogeneity has been poorly analyzed. Therefore, we compared molecular subtype classifications between TC and TNI regions within the primary tumors as well as the overlap of subtypes between the primary tumors and corresponding positive LNs. We observed a relatively high intratumor subtype concordance of 81% with the highest overlaps for Ba/Sq and GU subtypes and the lowest stability in the Sc/Ne group. Common subtype differences within the same primary tumor were detected between the GU and Uro subtypes.

Comparing the primary and LN metastatic sites, we observed an overall consistency of 57%. These subtype differences were mainly driven by shifts from Uro to GU. Moreover, the GU subtype also exhibited lower stability. In the comparable Lund study, the authors detected a lower molecular subtype heterogeneity, with an overlap of 82% between the primary and LN metastatic sites. In the discordant cases, most primary tumors were identified as Ba/Sq subtype (58%), while the corresponding LNs were identified as GU or Uro subtypes. Intratumor heterogeneity was observed in half of these discordant cases [28]. In contrast, in our study, the Ba/Sq subtype exhibited high stability both within the tumors and between the primary tumors and matched positive LNs.

Some driver mutations and therapeutic targets are unevenly distributed among different subtypes, suggesting the potential of molecular subtyping in therapeutic decision‐making. *EGFR* gain is enriched in Ba/Sq, while *ERBB2* amplification is more common in luminal unstable/GU subtypes [2, 29]. Interestingly, *ERBB2*/HER2 amplification was found to be associated with NAC response of MIBC patients, which is characteristic for the GU subtype [2, 30], and confirm our observation of greater CTx benefits among GU patients. Since HER2 is a promising target for both anti‐HER2 antibody and HER2‐directed antibody drug conjugate (ADC) treatments, GU tumors may benefit from these targeted therapies. This is especially relevant considering the agnostic FDA‐approval of trastuzumab deruxtecan in previously treated patients with metastatic HER2‐positive solid tumors. In addition, the GU subtype was also associated with higher ICI response rate, probably due to the presence of higher tumor mutation burden [31]. *FGFR3* amplification is enriched in the luminal papillary subtype (overlapping with the Lund Taxonomy Uro) and *FGFR3* alterations are associated with better response to NAC, aligning with our present observation of enhanced OS benefits from CTx [32]. The higher rate of *FGFR3* alterations in the luminal papillary/Uro subtype implies potential benefits from targeted *FGFR*‐inhibitor therapy, which is already recommended for molecularly selected metastatic MIBC patients with susceptible *FGFR2/3*‐gene fusions or *FGFR3* mutations [15]. Finally, Enfortumab vedotin, a nectin‐4‐targeting ADC has been approved for metastatic MIBC, both as third‐line monotherapy and recently in combination with pembrolizumab in the first‐line setting [33, 34]. Nectin‐4 was shown to be more abundantly expressed in luminal compared to basal tumors [35].

This study has some limitations by its retrospective nature and limited sample size for less frequent molecular subtypes. Although the study involved staining 1,508 cores for 13 IHC markers, with four cores per patient taken from different tumor regions – central tumor and TNI – plus two additional cores for cases with positive LNs, this limited sample size per patient may limit the accuracy of assessing tumor heterogeneity. The lack of radiologically or pathologically measurable lesions in the adjuvant setting represents a further limitation. However, patient selection in the adjuvant setting is based on definitive pathological staging, which is an advantage. Additional strengths of the study include testing the 13 IHC marker‐based Lund Taxonomy classification method in a large MIBC (RC and AC) cohort and assessing tumor heterogeneity both within the primary tumor and between primary tumor and corresponding LN metastasis. However, further reduction of the 13 markers is needed to make the Lund Taxonomy suitable for routine clinical use.

In the present study, applying a 13 IHC marker‐based Lund Taxonomy to a large MIBC cohort, we have confirmed for the first time that GU tumors derive greater benefit from AC. Furthermore, our data show relatively low subtype heterogeneity within the tumor, suggesting subtype stability, which provides robustness for molecular classification and as well as for its clinical applications.

## Author contributions statement

CO: writing‐original draft, data analysis and visualization. OS, GGK, JR, AK, ES and LS: data analysis and methodology. BH: resources, supervision, writing‐review and editing. UK, MV, CN and PN: writing‐review and editing. HR: conceptualization, methodology, data analysis, writing‐review and editing. TS: conceptualization, methodology, funding acquisition, data analysis and writing‐original draft.

## Supporting information


Supplementary methods and results
**Figure S1.** Expression of the 13 protein markers used for molecular subtype classification according to the Lund Molecular Taxonomy
**Figure S2.** Protein expression of the 13 IHC markers in the tumor central and tumor‐normal interface regions, and positive lymph nodes
**Figure S3.** Overall and radiographic progression‐free survival analyses in different treatment cohorts
**Figure S4.** Survival analyses of low and high age groups
**Figure S5.** Overall survival stratified by treatment in different molecular subtype groups
**Table S1.** Antibodies used for the molecular subtyping and details of immunohistochemical data for the 13 markers
**Table S2.** Association between molecular subtypes and clinical parameters
**Table S3.** Cox univariable analysis for overall survival and radiographic progression‐free survival

## Data Availability

Immunohistochemical data generated during this study are included in the article and in its supplementary information files. Patient‐level data are available on request from the corresponding author.
