# Supplementary material for: Immunohistochemistry‐based molecular subtypes of urothelial carcinoma derive different survival benefit from platinum chemotherapy
Source: J Pathol Clin Res. 2025 Jan 16;11(1):e70017. doi: 10.1002/2056-4538.70017 (PMC11736421; doi:10.1002/2056-4538.70017)
Supplement: Supplementary file 1 — Supplementary methods and results Figure S1. Expression of the 13 protein markers used for molecular subtype classification according to the Lund Molecular Taxonomy Figure S2. Protein expression of the 13 IHC markers in the tumor central and tumor‐normal interface regions, and positive lymph nodes Figure S3. Overall and radiographic progression‐free survival analyses in different treatment cohorts Figure S4. Survival analyses of low and high age groups Figure S5. Overall survival stratified by treatment in different molecular subtype groups Table S1. Antibodies used for the molecular subtyping and details of immunohistochemical data for the 13 markers Table S2. Association between molecular subtypes and clinical parameters Table S3. Cox univariable analysis for overall survival and radiographic progression‐free survival [file CJP2-11-e70017-s001.zip › cjp270017-sup-0001-SuppMatMeth,FiguresS1-S5,TablesS1-S3.pdf]

Immunohistochemistry-based molecular subtypes of urothelial carcinoma derive different survival benefit from platinum chemotherapy

C Olah *et al*. *J Pathol Clin Res* https://doi.org/10.1002/2056-4538.70017

Supplementary methods and results

Supplementary Figures S1–S5

Supplementary Tables S1–S3 (see separate Excel file for Table S1)

Reference numbers refer to the list in the main paper.

Supplementary methods and results

IHC analysis and molecular subtyping

Thirteen protein markers were stained and evaluated based on the Molecular Taxonomy method as described by the Lund research group [14]. The intensity and/or percentage of protein markers were determined as follows: the intensity was determined from 0-3 (negative, low, moderate, high), and the percentage was scored on a scale of 0 to 100% divided by 10 (0-10). Then, the intensity and percentage were multiplied to obtain the tumor-cell score. The cores were averaged from the different regions (tumor central (TC), tumor-normal interface (TNI), positive lymph node (LN+)). The tumor-cell scores were normalized by dividing the obtained scores by the maximum value of the range. This normalization process was conducted to ensure comparability among the evaluation values determined by percentage or intensities. Then, the basal/squamous (Ba/Sq), mesenchymal-like (Mes), small-cell/neuroendocrine-like (Sc/Ne), urothelial-like (Uro), and genomically unstable (GU) scores were determined based on the normalized tumor-cell scores of predefined markers. If the Ba/Sq, Mes or Sc/Ne score were higher than 0.6, the sample was identified as Ba/Sq or Mes or Sc/Ne, depending which scores were the highest. The remaining samples were identified as Uro if the Uro score was higher than 0.6, and as GU if the Uro score was lower than 0.6.

Cox univariable analysis

In the RC cohort, higher stage and the presence of positive LNs at RC were associated with shorter OS ($p=0.002$ and $p<0.001$, respectively). Both parameters remained significant risk factors for OS in the multivariable analysis (pT3/4: HR: 1.709, 95% CI: 1.025-2.848, $p=0.004$; LN+: HR: 2.707, 95% CI: 1.640-4.466, $p<0.001$). A higher progression rate tended to be observed among patients of older age and among females ($p=0.052$, $p=0.086$, respectively) (supplementary material, Table S3A,B).

Molecular subtype classification using a further reduced IHC marker set

While IHC-based assays with few markers can be easily integrated into clinical routine, staining for 13 protein markers for molecular subtyping can still be challenging and limits the applicability of the method. Therefore, efforts have been directed towards the elaboration of molecular subtype classification based on few (typically 2-4) IHC targets. Hardy *et al.* aimed to simplify the Lund IHC Taxonomy by reducing the marker set to 2-3 proteins [20]. Two simply models were suggested; Model 1 was used GATA3, KRT5, and p16 and identified Uro, GU, and Ba/Sq subtypes with a total accuracy of 78%, while Model 2 evaluated KRT14

and RB1 achieving 85% accuracy comparing to the 13-protein-based classification (Lund Taxonomy).

As the LundTax IHC 13-marker set applied in this study includes the markers of the simplified method mentioned above, we were able to assess their overlap with the LundTax classification as well as their prognostic and predictive performance (supplementary material, Figure S4A). In our samples, Model 1 achieved an overall overlap of 76% with the original LundTax subtypes. The Ba/Sq subtype exhibited the highest overlap (84%), followed by the GU (76%) and Uro (65%). The main cause of the discrepancy is the lower discriminative effect of the reduced marker set between the Uro and the GU subtypes as Model 1 identified 27% of LundTax-Uro tumors as GU, and 18% of the LundTax-GU as Uro. This low discriminative value of Model 1 between the Uro and GU subtypes may explain our observation that the use of platinum therapy provided OS benefit not only in the GU – like in the LundTax classified evaluation - but also in the Uro subtypes ($p=0.004$ and $p<0.001$, respectively) (supplementary material, Figure S4B).

Model 2 achieved a total overlap of 73% with the original LundTax subtypes. The GU subtype showed the highest overlap (80%), followed by the Ba/Sq (77%) and the Uro subtype (51%). The lower overlap in case of Uro subtype was again related to the lower discriminative effect of the simplified marker set between Uro and GU primarily because many of these tumors were classified as GU (47%) by Model 2. The survival analysis according to Model 2 showed that adjuvant chemotherapy was associated with longer OS only in the GU subtype ($p=0.002$) (supplementary material, Figure S4C).

Overall, the use of platinum-based AC was consequently associated with improved OS in the GU subtypes as classified by all the three classifier methods (Lund Taxonomy, Model 1, Model 2). In contrast, the OS in Ba/Sq tumors as classified by the 13 marker LundTax method proved to be similar between platinum-treated (AC) and untreated (RC) patient cohorts, suggesting that patients with Ba/Sq tumor do not benefit from AC. However, if the classification was performed by using the simplified marker set (Model 1 and Model 2) an insignificant trend towards OS benefit of AC-treated patients could be observed also in the Ba/Sq subtypes, suggesting that the simplified models show rather prognostic then platinum-predictive value. Overall, both Model 1 and Model 2 demonstrated the ability to classify samples into three subtypes (Uro, GU and Ba/Sq) with a relatively large overlaps (76% and 73%, respectively) with the 13-protein LundTax classification, however they showed poorer efficacy in differentiating between the GU and Uro subtypes. Furthermore, these data show

that molecular subtyping based on only 2-3 markers resulted in a trend towards reduced predictive value for platinum-based CTx.

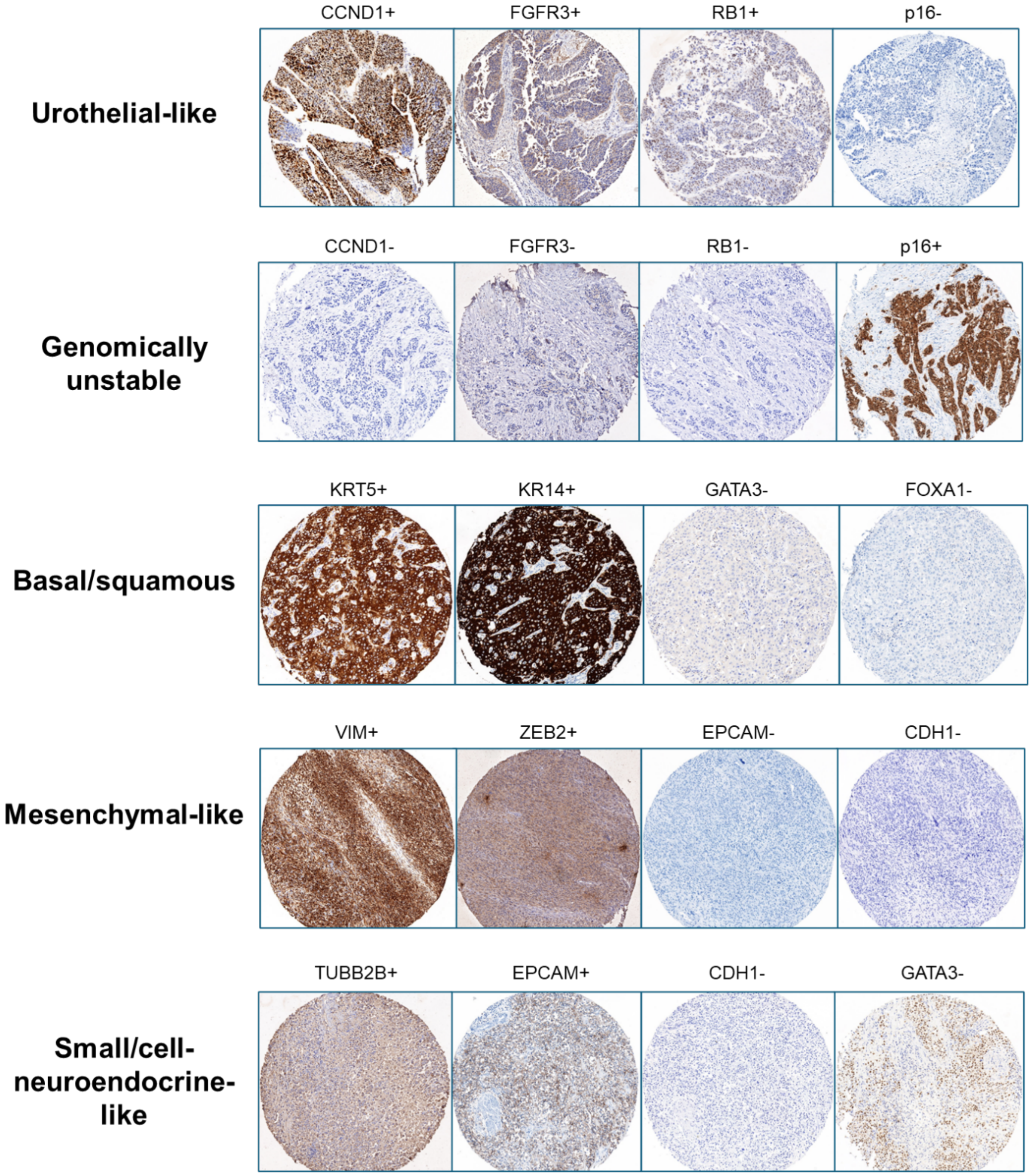

Figure S1. Expression of the 13 protein markers, used for molecular subtype classification according to the Lund Molecular Taxonomy, demonstrating distinct patterns characteristic of the five different molecular subtypes. The immunohistochemical stainings are presented at 10x magnification. Plus indicates strong staining intensity, while negative indicates negative or weak staining.

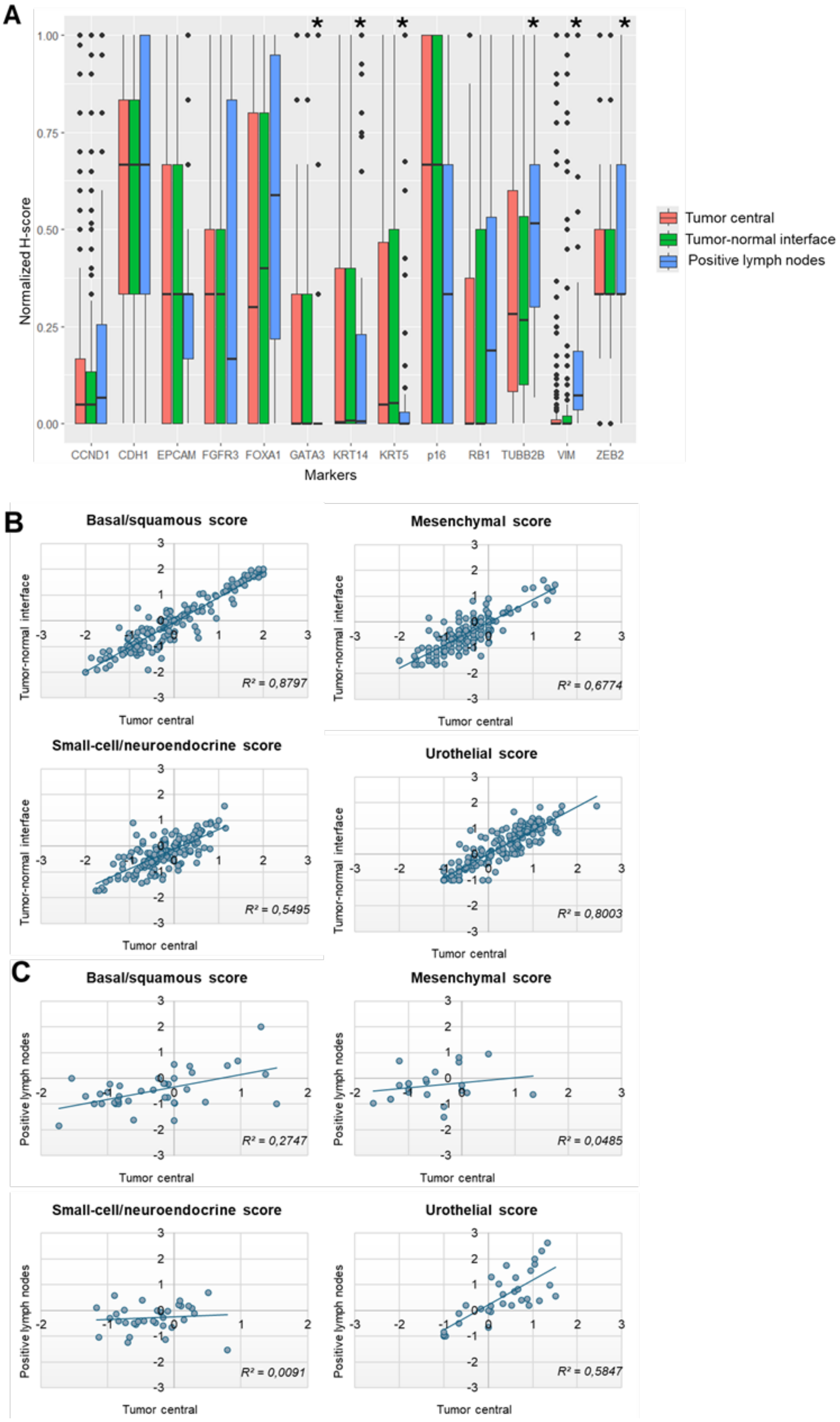

Figure S2. Protein expression of the 13 IHC markers in the tumor central and tumor-normal interface regions, and positive lymph nodes. (A) Protein expression of the 13 IHC markers in the tumor central and tumor-normal interface regions, and positive lymph nodes. Significant differentiations between primary tumors and positive lymph nodes are marked with stars. Correlation between different scores used for Lund Taxonomy, calculated from the expression of the 13 protein markers in (B) the tumor central and the tumor-normal interface comparison and (C) in the tumor central region and lymph nodes comparison.

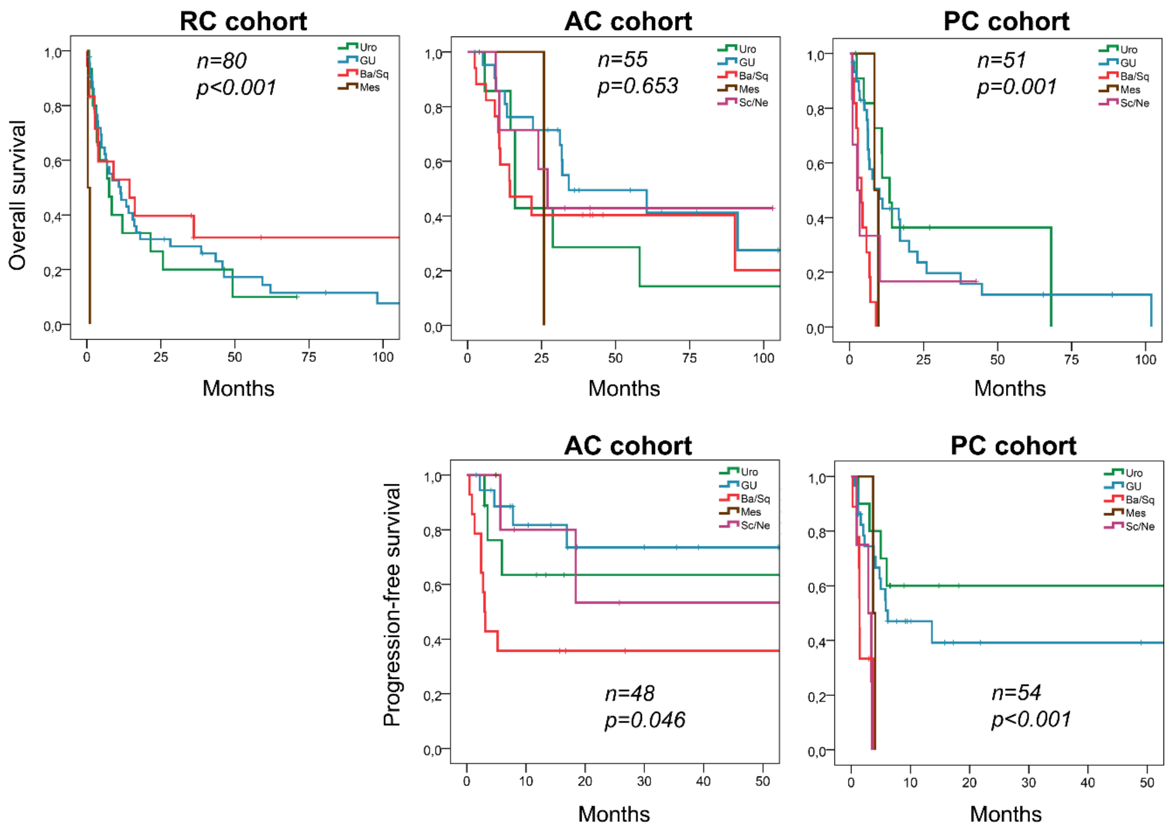

Figure S3. Overall and radiographic progression-free survival analyses in different treatment cohorts. AC: adjuvant chemotherapy, PC: palliative chemotherapy, RC: radical cystectomy, Ba/Sq: Basal/squamous, GU: Genomically unstable, Mes: Mesenchymal-like, Sc/Ne: Small-cell/neuroendocrine-like, Uro: Urothelial-like.

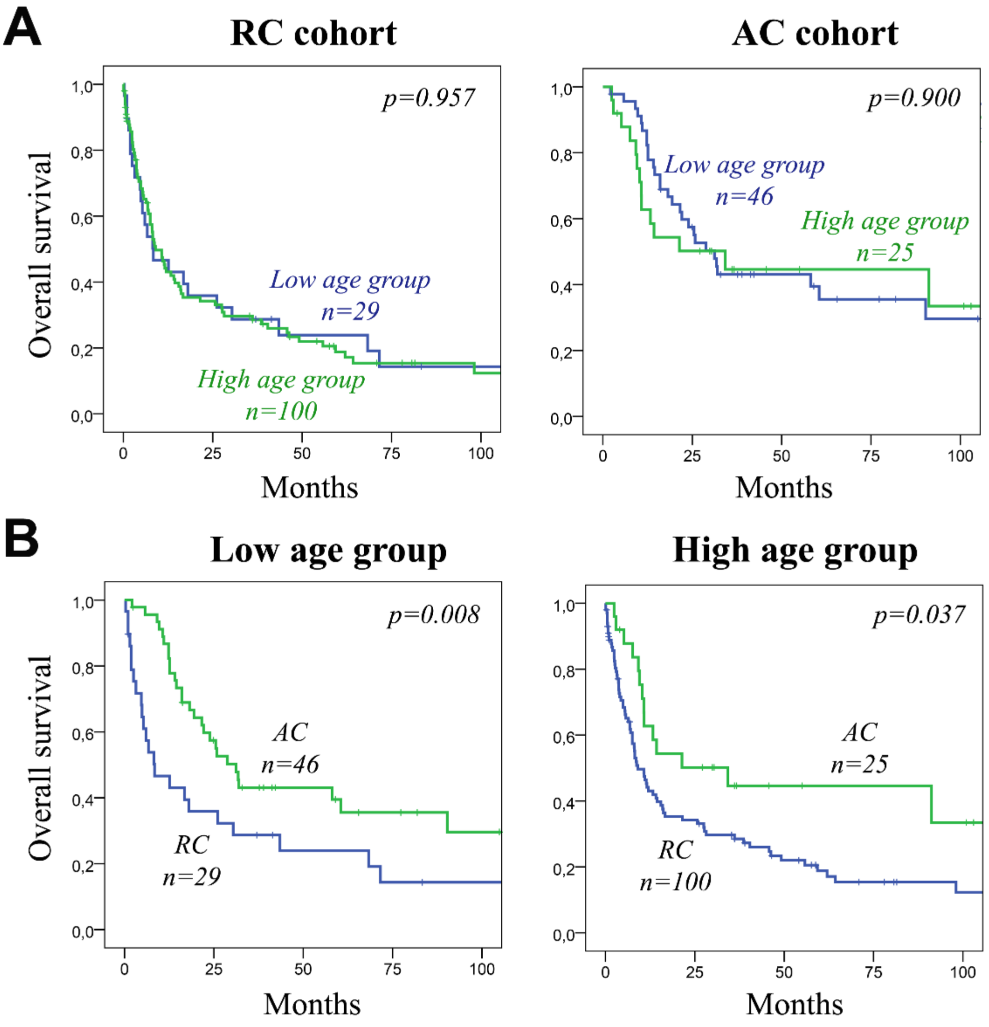

Figure S4. Survival analyses (A) between low (≤65 years) and high age (>65 years) groups in different treatment cohorts, and (B) between different treatment groups in age-stratified patient groups. RC cohort: solely radical cystectomy (RC)-treated patients, AC cohort: adjuvant chemotherapy (AC)-treated patients.

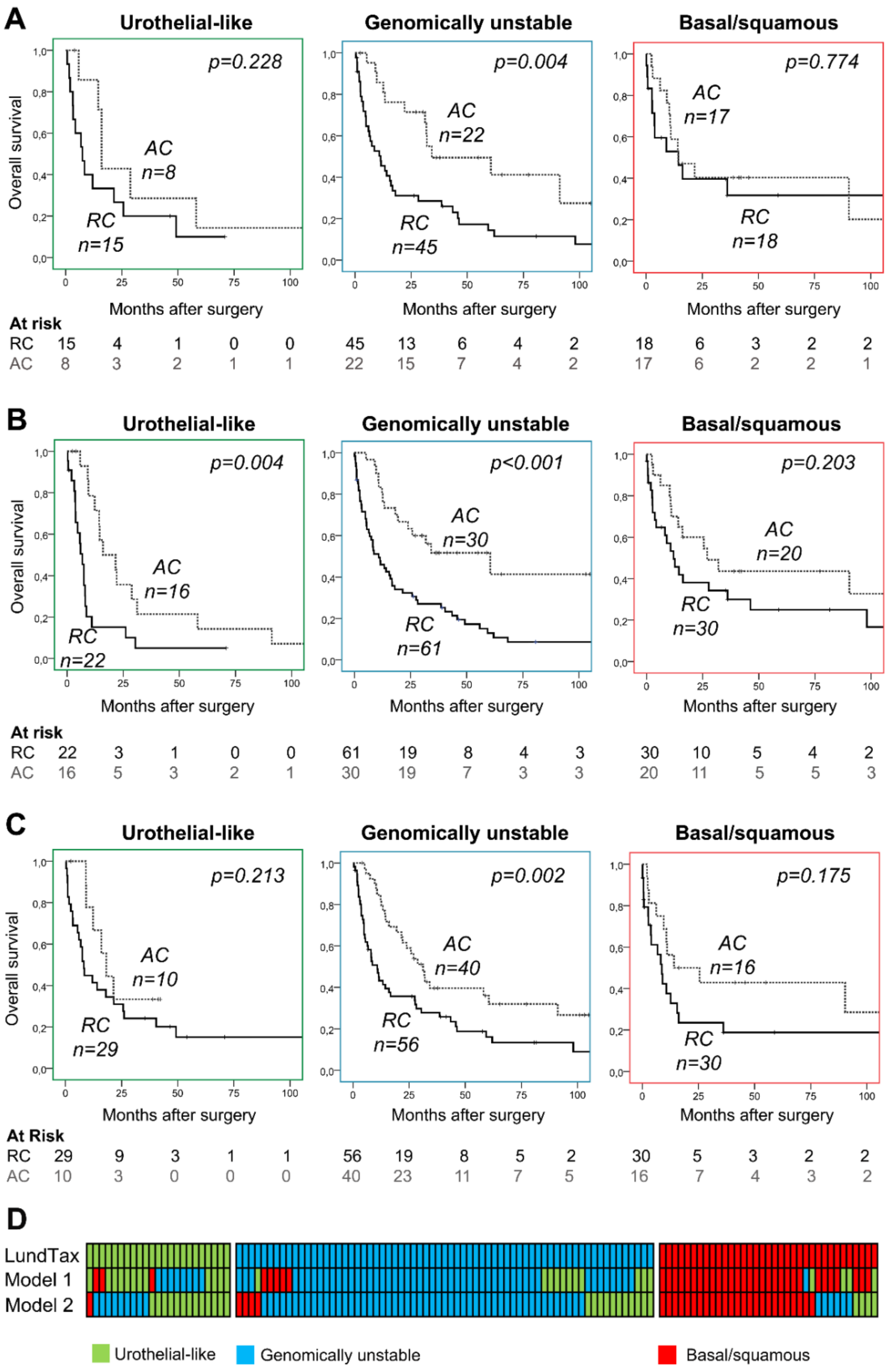

Figure S5. Overall survival stratified by treatment (RC vs AC) in different molecular subtype groups determined by (A) Lund Taxonomy using 13 markers, (B) Model 1 using 3 markers, and (C) Model 2 using 2 markers. (D) Overlaps of tumor samples as determined by different molecular subtype classifiers. AC: adjuvant chemotherapy, RC: radical cystectomy, LundTax: Lund Taxonomy.

Supplementary Tables

Table S1. Antibodies used for the molecular subtyping and details of immunohistochemical data for the 13 markers. Please see the separate Excel file.

Table S2. Association between molecular subtypes and clinical parameters by Chi^2 test. AC: adjuvant chemotherapy, CTx: chemotherapy, PC: palliative chemotherapy, Mol: Molecular, RC: radical cystectomy, LN: lymph node, Ba/Sq: Basal/squamous, GU: Genomically unstable, Sc/Ne: Small-cell/neuroendocrine-like, Uro: Urothelial-like.

**Clinical parameters**	**Age (baseline)**			**Sex**			**Stage at RC**			**LN metastases at RC**			**Distant metastases at RC**		
	≤ 65	> 65		Male	Female		pT2	pT3-4		LN0/LNx	LN+		M0/Mx	M+	
	n (%)	n (%)	P	n (%)	n (%)	P	n (%)	n (%)	P	n (%)	n (%)	P	n (%)	n (%)	P
**Whole cohort**															
Mol. subtypes (ref. Ba/Sq)	16 (35)	30 (65)		27 (59)	19 (41)		3 (7)	41 (93)		30 (65)	16 (35)		-	-	-
Uro	14 (40)	21 (60)	0.630	28 (80)	7 (20)	**0.042**	6 (20)	24 (80)	0.089	19 (54)	16 (46)	0.319	-	-	-
GU	40 (41)	58 (59)	0.489	77 (79)	21 (21)	**0.013**	9 (11)	75 (89)	0.473	66 (67)	33 (32)	0.864	-	-	-
Sc/Ne	8 (53)	7 (47)	0.202	12 (80)	3 (20)	0.136	4 (27)	11 (73)	**0.043**	11 (73)	4 (27)	0.561	-	-	-
**RC cohort**															
Mol. subtypes (ref. Ba/Sq)	1 (6)	17 (94)		12 (67)	6 (33)		0 (0)	18 (100)		13 (72)	5 (28)		0	0	
Uro	2 (13)	13 (87)	0.439	13 (87)	2 (13)	0.182	2 (13)	13 (87)	0.110	8 (53)	7 (47)	0.261	0	0	-
GU	8 (18)	37 (82)	0.210	36 (80)	9 (20)	0.262	0 (0)	45 (100)	-	31 (69)	14 (31)	0.795	0	0	-
**AC cohort**															
Mol. subtypes (ref. Ba/Sq)	9 (56)	7 (44)		10 (59)	7 (41)		2 (12)	15 (88)		10 (59)	7 (41)		0	0	-
Uro	6 (75)	2 (25)	0.371	7 (88)	1 (13)	0.152	2 (25)	6 (75)	0.400	3 (38)	5 (63)	0.319	0	0	-
GU	14 (64)	8 (36)	0.646	15 (68)	7 (32)	0.546	2 (9)	20 (91)	0.786	9 (41)	13 (5)	0.267	0	0	-
Sc/Ne	4 (67)	2 (33)	0.658	5 (71)	2 (29)	0.088	3 (43)	4 (57)	0.146	6 (86)	1 (14)	0.308	0	0	-
**PC cohort**															
Mol subtypes (ref. Ba/Sq)	6 (55)	5 (45)		5 (45)	6 (55)		1 (11)	8 (89)		7 (64)	4 (36)		5 (45)	6 (55)	
Uro	6 (50)	6 50)	0.827	8 (67)	4 (33)	0.305	2 (29)	5 (71)	0.375	8 (67)	4 (33)	0.879	6 (50)	6 (50)	0.827
GU	17 (55)	14 (45)	0.987	26 (84)	5 (16)	**0.013**	7 (41)	10 (59)	0.114	26 (81)	6 (19)	0.233	19 (59)	13 (41)	0.759
Sc/Ne	2 (29)	5 (71)	0.280	6 (86)	1 (14)	0.087	3 (43)	4 (57)	0.146	6 (86)	1 (14)	0.308	5 (29)	2 (71)	0.280

Table S3. Cox univariable analysis for (A) overall survival and (B) radiographic progression-free survival. AC: adjuvant chemotherapy, CTx: chemotherapy, PC: palliative chemotherapy, LN: lymph node, M: distant metastasis, RC: radical cystectomy, Ba/Sq: Basal/squamous, GU: Genomically unstable, Mes: Mesenchymal-like, Sc/Ne: Small-cell/neuroendocrine-like, Uro: Urothelial-like.

**A Overall survival**			**RC cohort**			**AC cohort**			**PC cohort**		
**variables**			HR	95% CI	P	HR	95% CI	P	HR	95% CI	P
Age	> 65	ref. ≤ 65	1.123	0.786-1.605	0.525	1.080	0.521-2.239	0.836	1.031	0.586-1.815	0.915
Sex	Female	ref. male	0.922	0.560-1.519	0.750	1.060	0.521-2.157	0.872	1.239	0.639-2.402	0.526
Stage	pT3-4	ref. pT2	2.138	1.315-3.475	**0.002**	0.603	0.245-0.468	0.272	0.890	0.433-1.830	0.752
Metastases	LN+	ref. LN0/LNx	3.240	2.025-5.184	**<0.001**	1.483	0.745-2.950	0.262	0.903	0.462-1.765	0.765
	M+	ref. M0/Mx	-			-			0.942	0.534-1.663	0.838
Mol. subtype	Uro	ref. Ba/Sq	1.030	0.544-1.951	0.928	0.883	0.329-2.372	0.805	0.110	0.029-0.418	**0.001**
	GU	ref. Ba/Sq	0.970	0.742-1.267	0.822	0.796	0.527-1.202	0.277	0.500	0.333-0.751	**0.001**
	Mes	ref. Ba/Sq	1.026	0.755-1.394	0.871	1.019	0.608-1.708	0.943	0.638	0.377-1.079	0.094
	Sc/Ne	ref. Ba/Sq	-	-	-	0.927	0.737-1.167	0.520	0.923	0.730-1.166	0.501

**B Radiographic progression-free survival**			**RC cohort**			**AC cohort**			**PC cohort**		
**variables**			HR	95% CI	P	HR	95% CI	P	HR	95% CI	P
Age at CTx	> 65	ref. ≤ 65	-	-	-	2.101	0.848-5.203	0.109	1.997	0.994-4.012	0.052
Sex	Female	ref. male				0.581	0.193-1.755	0.336	1.964	0.909-4.164	0.086
Stage at CTx	pT3-4	ref. pT2	-	-	-	2.698	0.358-20.316	0.335	1.150	0.498-2.657	0.743
Metastases	LN+	ref. LN0/LNx	-	-	-	1.088	0.493-2.694	0.855	1.679	0.824-3.420	0.154
	M+	ref. M0/Mx	-	-	-	-		0.277	1.753	0.870-3.529	0.116
Mol. subtype	Uro	ref. Ba/Sq	-	-	-	0.360	0.097-1.336	0.127	0.060	0.007-0.519	**0.011**
	GU	ref. Ba/Sq	-	-	-	0.502	0.289-0.870	**0.014**	0.598	0.372-0.961	**0.034**
	Mes	ref. Ba/Sq	-	-	-	0.455	0.031-6.578	0.563	0.673	0.392-1.153	0.150
	Sc/Ne	ref. Ba/Sq	-	-	-	0.819	0.601-1.116	0.206	0.961	0.696-1.327	0.809
